# Comparative analysis of anti-polyglutamine Fab crystals grown on Earth and in microgravity

**DOI:** 10.1107/S2053230X16014011

**Published:** 2016-09-22

**Authors:** Gwen E. Owens, Danielle M. New, Alejandra I. Olvera, Julia Ashlyn Manzella, Brittney L. Macon, Joshua C. Dunn, David A. Cooper, Robyn L. Rouleau, Daniel S. Connor, Pamela J. Bjorkman

**Affiliations:** a Division of Biology and Biological Engineering, California Institute of Technology, 1200 East California Boulevard, Pasadena, CA 91125, USA; b Graduate Option in Biochemistry and Molecular Biophysics, California Institute of Technology, 1200 East California Boulevard, Pasadena, CA 91125, USA; c UCLA–Caltech Medical Scientist Training Program, Los Angeles, CA 90095, USA; d Division of Chemistry and Chemical Engineering, California Institute of Technology, 1200 East California Boulevard, Pasadena, CA 91125, USA; e Center for Biophysical Sciences and Engineering, University of Alabama at Birmingham, 1025 18th Street South, Birmingham, AL 35294, USA

**Keywords:** Huntington’s disease, International Space Station, microgravity, polyglutamine, X-ray crystallography, huntingtin, crystallization

## Abstract

Huntington’s disease is one of nine neurodegenerative diseases caused by a polyglutamine (polyQ)-repeat expansion. An anti-polyQ antigen-binding fragment, MW1 Fab, was crystallized both on Earth and on the International Space Station, a microgravity environment where convection is limited. Once the crystals returned to Earth, the number, size and morphology of all crystals were recorded, and X-ray data were collected from representative crystals. The results generally agreed with previous microgravity crystallization studies. On average, microgravity-grown crystals were 20% larger than control crystals grown on Earth, and microgravity-grown crystals had a slightly improved mosaicity (decreased by 0.03°) and diffraction resolution (decreased by 0.2 Å) compared with control crystals grown on Earth. However, the highest resolution and lowest mosaicity crystals were formed on Earth, and the highest-quality crystal overall was formed on Earth after return from microgravity.

## Abstract 2

Microgravity was used in an attempt to define the crystal structure of the polyQ repeat of huntingtin alone or bound to MW1, an anti-polyQ Fab. While huntingtin was not crystallized in the experiments, analysis of microgravity-grown and Earth-grown MW1 Fab crystals showed that, on average, microgravity-grown crystals of MW1 Fab showed an increase in size and an improvement in resolution and mosaicity when compared with Earth-grown crystals in one space group, in agreement with data published for other proteins, although the highest overall resolution X-ray data in our experiments were obtained from a crystal grown on Earth.

## 1. Introduction   

Huntington’s disease (HD) is a progressive, late-onset neurodegenerative disease characterized by neuronal death resulting in choreiform movements, cognitive decline and behavioral abnormalities (Ross *et al.*, 2014 ▸). There is currently no disease-modifying treatment or cure for HD (Skotte *et al.*, 2014 ▸). HD is found in individuals with an abnormally expanded N-terminal polyglutamine (polyQ) repeat in huntingtin, a 350 kDa protein of unknown function. The length of this polyQ tract influences the mechanisms of aggregation and associated binding kinetics, with an increasing propensity for aggregation with increasing polyQ tract length (Thakur *et al.*, 2009 ▸). HD is completely penetrant when the polyQ repeat expands beyond a 41-glutamine threshold. However, the mechanism underlying this transition is unclear, and the relationship between polyQ-mediated aggregation, cellular toxicity and HD symptoms has not been well characterized. The structure of polyQ with >41 glutamine residues has been suggested to adopt a new β-sheet conformation (Nagai *et al.*, 2007 ▸) or a random-coil conformation (Vitalis *et al.*, 2008 ▸). Several glutamines in nonpathologic polyQ stretches of huntingtin have previously been crystallized as part of a maltose-binding protein (MPB) fusion protein at 3.5 Å resolution (Kim *et al.*, 2009 ▸; PDB entries 3io4, 3io6, 3ior, 3iot, 3iou, 3iov and 3iow). In this series of structures, the modeled polyQ region was conformationally flexible and was affected by the conformation of nearby residues. However, in the absence of an X-ray crystal structure of the entire polyQ-repeat region, the nature of the expanded polyQ region and the interactions between mutant polyQ and other proteins are unclear.

The polyQ repeat in huntingtin is recognized by several monoclonal anti-polyQ antibodies, including MW1-6 (Ko *et al.*, 2001 ▸), 3B5H10 (Brooks *et al.*, 2004 ▸), 1C2 (Lescure *et al.*, 1994 ▸; Trottier *et al.*, 1995 ▸) and 1F8 (White *et al.*, 1997 ▸; Persichetti *et al.*, 1999 ▸), which has been reported to be similar to 1C2 (White *et al.*, 1997 ▸). The X-ray crystal structures of the 3B5H10 Fab (PDB entries 3s96 and 4dcq; Peters-Libeu *et al.*, 2005 ▸, 2012 ▸), the 1C2 Fab (PDB entries 4isv and 4jj5; Klein *et al.*, 2013 ▸) and the MW1 Fv (PDB entries 2gsg, 2otu and 2otw; Li *et al.*, 2007 ▸) are structurally homologous (Klein *et al.*, 2013 ▸). While each of these antibodies has a different apparent affinity for polyQ, 1C2, 3B5H10 and MW1 all have lambda light chains, homologous sequences and strong structural similarity (Klein *et al.*, 2007 ▸). Two recent papers reported the measurement of huntingtin protein in the cerebrospinal fluid of patients with HD using the MW1 antibody as one of a pair of antibodies in immuno­precipitation-flow cytometry (Southwell *et al.*, 2015 ▸) or FRET (Ross *et al.*, 2014 ▸).

Information about the three-dimensional structure of MW1 and its interactions with mutant huntingtin could assist in the development of polyQ length-based methods for quantitation of huntingtin in patients with HD in clinical trials (Zuccato *et al.*, 2010 ▸). X-ray crystal structures of the MW1 fragment variable (Fv; the variable heavy and variable light domains; V_H_ and V_L_) alone (PDB entry 2gsg) and in complex with a GQ_10_G peptide (PDB entries 2otu and 2otw) (Li *et al.*, 2007 ▸) demonstrated that the polyQ epitope could adopt a linear and extended conformation within a shallow groove of the MW1 Fv and also demonstrated that the binding epitope for MW1 encompasses ∼10 glutamines. Major structural changes occurred in MW1 Fv upon polyQ binding, including movement of amino acids in the third complementarity-determining regions (CDRs) of the heavy-chain and light-chain variable domains (CDRH3 and CDRL3) to allow hydrogen-bond formation between the antigen-binding site and polyQ.

We previously attempted to crystallize huntingtin alone and in complex with Fabs (antigen-binding fragments) of anti-polyQ antibodies, including MW1 Fab. While stable complexes of Fabs with the polyQ-containing N-terminal domain of huntingtin with 16, 25, 39 and 46 glutamines (HD-16Q, HD-25Q, HD-39Q and HD-46Q) formed in solution (Owens *et al.*, 2015 ▸), no crystals of huntingtin or of a Fab–huntingtin complex could be obtained. Crystallization of the polyQ stretch of huntingtin is particularly challenging owing to polyQ length-dependent aggregation (Temussi *et al.*, 2003 ▸).

Reduced-gravity environments may improve crystal formation for proteins such as huntingtin that have a propensity to form a disordered aggregate at high concentrations owing to reduction in buoyancy-driven convection. In low-convection environments, mass transport is primarily driven by diffusion. Aggregates diffuse more slowly than monomers; therefore, monomers may have greater access to the surface of the growing crystal than aggregates in microgravity (McPherson & DeLucas, 2015 ▸). Microgravity has led to improved crystal volume and quality for several proteins including insulin (Borgstahl *et al.*, 2001 ▸; Dong *et al.*, 1999 ▸), a protein that has been used as a model of amyloid formation (Ivanova *et al.*, 2009 ▸), and PPG10, a collagen-like protein with a polyproline region similar to that flanking the polyQ region of huntingtin (Vergara *et al.*, 2002 ▸). To gain further insight into the interaction between anti-polyQ Fabs and the polyQ repeat of huntingtin, we conducted protein-crystallization experiments on the International Space Station (ISS); this provided an environment where protein crystals could grow undisturbed for several months in a microgravity environment. Crystallization studies in microgravity and parallel ground-control tests were designed to examine whether we could generate high-quality crystals of polyQ proteins or crystals of a polyQ-containing protein in complex with anti-polyQ Fabs. We were unable to generate crystals of polyQ-containing proteins on the ISS or in ground controls, but present here a comparative analysis of MW1 Fab crystals grown in microgravity and on Earth.

## 2. Methods   

### 2.1. Protein expression and purification   

MW1 Fab was purified as described previously (Owens *et al.*, 2015 ▸). Briefly, MW1 Fab was prepared by papain cleavage of MW1 IgG and protein A affinity chromatography (GE Healthcare, Little Chalfont, England), with further purification by size-exclusion chromatography (SEC; Superdex 200 10/300 GL). Purified protein was stored at 4°C in 50 m*M* Tris pH 8.0, 150 m*M* NaCl for up to three months. Other proteins used in our microgravity crystallization trials included human huntingtin exon 1-thioredoxin (TRX) fusion proteins (HD-16Q, HD-25Q, HD-39Q and HD-46Q; Owens *et al.*, 2015 ▸; Bennett *et al.*, 2002 ▸), GFP-huntingtin (Sabogal & Rio, 2010 ▸) and the Fab from an MW1-related antibody called 3B5H10 (Miller *et al.*, 2011 ▸). The GFP-huntingtin construct was a gift from Dr Robert Hughes (Buck Institute, Novato, California, USA). Each of these proteins was purified using Ni^2+^–NTA affinity chromatography (GE Healthcare) and SEC (Superdex 200 10/300 or 16/60), flash-frozen and stored at −80°C in 50 m*M* Tris pH 8.0, 150 m*M* NaCl, with the exception of GFP-huntingtin, which was stored at −80°C in 10 m*M* HEPES pH 7.5, 50 m*M* NaCl, 10% glycerol, 0.5 m*M* tris(2-carboxyethyl)­phosphine (TCEP), a reducing agent. Purified full-length huntingtin protein (Seong *et al.*, 2010 ▸) was a gift from Dr IhnSik Seong (Massachusetts General Hospital) and was stored at −80°C in 50 m*M* Tris pH 8.0, 100 m*M* NaCl. Protein concentrations were determined using 280 nm extinction coefficients of 78 310 *M* ^−1^ cm^−1^ (MW1 Fab), 80 830 *M* ^−1^ cm^−1^ (3B5H10 Fab), 14 180 *M* ^−1^ cm^−1^ (HD-16Q, HD-25Q, HD-39Q and HD-46Q) and 22 015 *M* ^−1^ cm^−1^ (GFP-huntingtin). Extinction coefficients were calculated based on the amino-acid sequence using *ProtParam* (Gasteiger *et al.*, 2005 ▸). A bicinchoninic acid (BCA) assay (Thermo Scientific, Rockford, Illinois, USA) was used to determine the total protein concentration of full-length huntingtin.

For crystallization of the MW1 Fab alone, the protein was concentrated to 7 mg ml^−1^ using a centrifugal filter (EMD Millipore, Darmstadt, Germany). For MW1 Fab/HD-39Q co-crystallization trials, purified MW1 Fab and HD-39Q were incubated in a 3:1 molar ratio for at least 1 h at 4°C. Crystallization conditions were optimized on Earth prior to microgravity experiments. Initial concentrations for all proteins are listed in Supplementary Table S1.

### 2.2. Vapor-diffusion crystallization   

**Figure 1:**
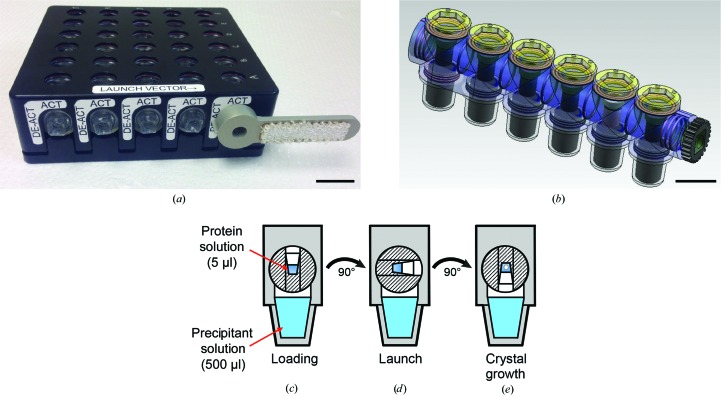
Schematics of the HDPCG device used for microgravity crystallization experiments. (*a*) Handheld HDPCG assembly, (*b*) HDPCG sample block, (*c*) HDPCG growth cell in loading configuration, (*d*) HDPCG growth cell in launch configuration, (*e*) HDPCG growth cell in microgravity crystal-growth configuration. The scale bar is 5 mm in length.

Vapor-diffusion protein crystal-growth experiments in microgravity were performed using the handheld High Density Protein Crystal Growth (HDPCG) hardware (Fig. 1 ▸ *a*) developed by the Engineering group at the Center for Biophysical Sciences and Engineering at the University of Alabama, Birmingham. The handheld HDPCG hardware was designed to reproduce a sitting-drop or hanging-drop crystallization experiment in a microgravity environment (DeLucas *et al.*, 2003 ▸), and it has been used to crystallize dozens of proteins on the Space Shuttle and ISS (Abd Rahman *et al.*, 2015 ▸; Krauspenhaar *et al.*, 2002 ▸; Ponassi *et al.*, 2011 ▸). Each aluminium handheld HDPCG hardware unit held five HDPCG sample blocks (Fig. 1 ▸ *b*), which were molded from Zeonor plastic. Each sample block consisted of six individual growth cells that each contained a single vapor-diffusion crystal-growth experiment (Fig. 1 ▸ *c*). Each growth cell was isolated by triple O-ring containment.

For the preparation of experiments prior to launch, 2.5 µl protein solution was mixed with an equal volume of precipitant solution and placed in a 5 µl well in a growth cell. A separate reservoir in the same growth cell was loaded with ∼500 µl precipitant solution at the desired final concentration. The precipitant solution was immobilized in the reservoir using an insert made of Chromex, a porous absorbent material composed of ultrahigh-molecular-weight polyethylene (Porex, Fairburn, Georgia, USA). A total of 120 crystallization experiments were set up: 60 for microgravity experiments and 60 replicates as ground controls. Initial conditions for all experiments are listed in Supplementary Table S1. After all solutions had been loaded, each well was checked to ensure there were no bubbles and the sample blocks were sealed. Ten HDPCG sample blocks total were loaded into two handheld HDPCG hardware units. To prevent movement or mixing of solutions prior to or during launch, the sample-block barrels were rotated 90° clockwise to ‘launch configuration’ (Fig. 1 ▸ *d*).

For experiment activation in orbit, an astronaut rotated the sample-block barrels another 90° clockwise using an Activation Tool (the silver object in Fig. 1 ▸ *a*) to establish an air path between the protein solution in the well and the precipitant solution in the reservoir (Fig. 1 ▸ *e*). After activation, the experiments were stored undisturbed on the ISS. Initially, the protein solution contained an insufficient concentration of precipitant for crystallization, but as water vaporized from the droplet and transferred to the reservoir, the precipitant concentration in the protein well increased to an optimal level for crystallization in some experiments. Before return to Earth, an astronaut resealed the experiment by using the Activation Tool to rotate the sample-block barrel counterclockwise 90° to ‘launch configuration’ to turn the protein inserts away from the precipitant reservoir.

### 2.3. Timeline   

**Figure 2:**
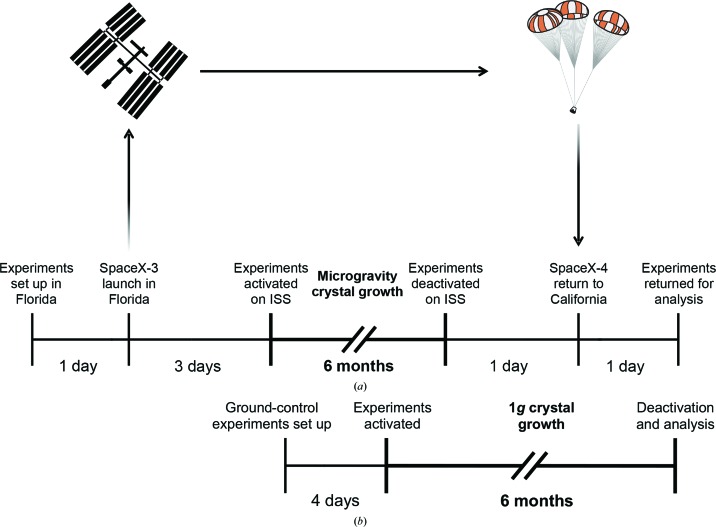
Timeline of (*a*) microgravity and (*b*) ground-control experiments.

All protein and precipitant stock solutions were prepared 7–52 d before launch. Proteins, precipitant solutions and other equipment were transported to the Space Station Processing Facility, Kennedy Space Center (KSC), Florida between 9 March and 10 April 2014 for transport on SpaceX CRS-3. Proteins were maintained at −20°C (HD-16Q, HD-25Q, HD-39Q, HD-46Q, GFP-huntingtin and full-length huntingtin) or 4°C (MW1 Fab and 3B5H10 Fab) during transport and storage. Before launch, flight HDPCG growth cells were prepared at 4°C in a cold room previously used for astronaut food storage. Owing to launch-vehicle delays immediately before two launches, all experimental materials were prepared repeatedly for launch prior to the successful launch on 18 April 2014. After each scrubbed launch, new inserts with fresh protein were loaded into the HDPCG apparatus to prevent protein degradation or aggregation. HDPCG sample filling and hardware integration was completed on 16 April 2014 (Fig. 2 ▸). The HDPCG hardware units were turned over to the ISS Cold Stowage team for integration on 17 April 2014 and were installed at 4°C on the same day in a Double Cold Bag, a Nomex bag with vacuum insulation panels for passive thermal insulation (Campana & Melendez, 2011 ▸). The phase-change material Ice Bricks were added to the Double Cold Bag to maintain a 4°C environment for the HDPCG hardware units prior to launch and during ascent to the ISS.

A total of 60 experimental crystallization trials in two handheld HDPCG hardware units were launched from Cape Canaveral, Florida in an unmanned Falcon-9 supply vehicle on 18 April 2014 at 15:25 Eastern Daylight Time (EDT). The HDPCG hardware units were maintained at 4°C in a Double Cold Bag during flight. The Dragon capsule mounted atop the Falcon-9 berthed with the ISS on 21 April 2014, the units were transferred to the ISS and activation was completed by Flight Engineer Steven Swanson at 06:50 EDT. After activation, the experiments were placed in a specialized refrigerator–freezer (Minus Eighty Laboratory Freezer for ISS, MELFI) set at 4°C. Crystals were allowed to grow undisturbed in microgravity at 4°C for exactly six months (183 d). The CASIS PCG hardware remained in the MELFI until just prior to unberth of the SpaceX-4 Dragon vehicle. HDPCG deactivation and transfer to a Double Cold Bag at 4°C for return was completed at 04:34 EDT on 24 October 2014 by Flight Engineer Reid Wiseman.

The deactivated experiments descended to Earth on 25 October 2014 in SpaceX-4. The Dragon capsule landed in the Pacific Ocean on 25 October 2014 at 15:39 EDT, and the experiments were handed over for transfer to Caltech in Pasadena, California at 22:35 EDT on 26 October 2014. A temperature of 4 ± 2°C was maintained during all transport operations. The HDPCG hardware units were stored and imaged, and crystals were harvested, in a 4°C room. There were no pre-flight, in-flight or post-flight anomalies.

### 2.4. Ground control and comparison studies   

The results of the microgravity crystallization experiments were evaluated on Earth using the best crystals that could be grown in identical conditions to, and using the same hardware as, the space-flight experiments, which we termed ‘ground controls’. Ground controls to replicate the conditions in the space-flight experiments were set up at 4°C at Caltech in HDPCG sample blocks with identical purified proteins, buffers and precipitant solutions as used for the microgravity payload. Similar delays between preparation and activation were used for the ground controls (Fig. 2 ▸), with a 7 d delay overall compared with flight experiments.

### 2.5. Crystal number, size and morphology analysis   

Immediately upon return from the ISS, bright-field images of all microgravity crystallization wells were manually taken at 40× magnification on an Olympus SZX16 microscope (Olympus Corporation, Tokyo, Japan) using a Canon DS126311 EOS Rebel camera (Canon, Tokyo, Japan). Crystals were found at the bottom of the wells; thus, the crystals were in focus at the same depth of field for all wells. The ground-control wells also were imaged, with an approximately 7 d delay. *Fiji* (Schindelin *et al.*, 2012 ▸) was used to measure the long axis and short axis of each crystal using the line tool. The area of each crystal was calculated both by multiplying the long and the short axis and by either the polygon selection tool or a freehand selection tool based on crystal shape. A MicroRuler (MiTeGen, Ithaca, New York, USA) was used to scale images in micrometres. Crystal number, morphology and visible area were recorded. Morphology was judged by the sharpness of the crystal edges and the shape of the crystal. Crystals were photographed again five months after return to Earth to evaluate changes in crystal size and morphology.

After crystals had been harvested for X-ray diffraction data collection, the remaining crystals were imaged with a Korima PRS-1000 UV microscope (Korima, Carson, California, USA) at 25°C to distinguish protein crystals from salt crystals based on tryptophan fluorescence under UV light. Representative bright-field and UV images of wells containing protein crystals are shown in Supplementary Figs. S1 and S2, respectively. Some small crystals seen with UV microscopy were not visible using bright-field microscopy; size and X-ray diffraction data were not collected for these crystals.

### 2.6. Crystallographic data collection and data-quality analysis   

Protein crystals were removed from the HDPCG sample wells and briefly soaked in mother-liquor solution supplemented with 7.5%, 15% and then 30% glycerol before flash-cooling in liquid nitrogen. X-ray diffraction data were collected from a total of 155 representative microgravity-grown and Earth-grown crystals on beamline 12-2 at the Stanford Synchrotron Radiation Lightsource (SSRL) using a PILATUS 6M pixel detector (Dectris) in top-up mode with an oscillation angle of 0.15°, λ = 0.98 Å and 500 mA ring current. Crystal-to-detector distances ranged from 270 to 800 mm. X-ray diffraction data sets were collected for 32 crystals.

X-ray diffraction data were unobtainable for some small crystals grown in microgravity owing to technical limitations. The collected data sets were indexed, integrated and scaled using *XDS*, a crystallographic data-processing program (Kabsch, 2010*a* ▸,*b* ▸).

Data quality was analyzed using *XDS* and the *PHENIX* crystallography package (Adams *et al.*, 2010 ▸). The overall resolution limits of each data set were estimated using *I*/σ(*I*) > 1.50 as well as CC_1/2_ (the correlation coefficient between two random halves of the data set; Karplus & Diederichs, 2012 ▸), where CC_1/2_ > 0.3. The average mosaicity was determined using the scaling program *AIMLESS* (Evans & Murshudov, 2013 ▸).

## 3. Results   

### 3.1. MW1 Fab crystals formed in microgravity had increased size and decreased abundance compared with crystals grown on Earth, while their morphology remained similar   

**Figure 3:**
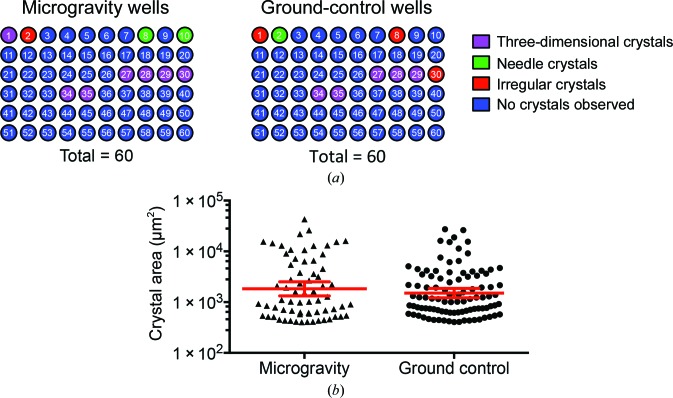
Comparison of crystal morphology and size. Each data point represents a one crystal.. Protein crystals grew in both microgravity and ground-control conditions. (*a*) Morphology of crystals and number of wells containing crystals. Wells 1–5 were set up with MW1 or 3B5H10 Fab, wells 6–10 with Fab + peptide, wells 11 and 12 with HD-16Q, wells 13–22 with Fab + HD-16Q, wells 23–36 with Fab + HD-39Q, wells 37–48 with HD-16Q, HD-25Q, HD-36Q or HD-46Q, wells 49–54 with huntingtin-GFP and wells 55–60 with full-length huntingtin. See Supplementary Table S1 for a complete description of the initial conditions. (*b*) Area of crystals greater than 400 µm^2^ grown in microgravity (*n* = 67) and on Earth (*n* = 97). Data shown are geometric means with 95% confidence intervals. The geometric mean is suitable for data that range over several orders of magnitude (West *et al.*, 2010 ▸).

Crystals formed in several wells both in microgravity and on Earth. All crystals formed in wells containing MW1 Fab alone or MW1 Fab with HD-39Q. No crystals were observed in any wells containing full-length huntingtin or GFP-huntingtin; instead, the presence of UV-fluorescent aggregate was noted in these wells. Crystals were observed in ten of 60 wells in the microgravity HDPCG wells and in nine of 60 wells in the ground-control HDPCG wells (Fig. 3 ▸ *a*, Table 1 ▸). Of the wells containing crystals, one was found only in the flown samples, *i.e.* there were no crystals in the corresponding ground-control well. This well contained needle crystals that did not diffract beyond 5 Å resolution. No crystals were observed in the ground-control wells without also being observed in the sample wells in the flown HDPCG growth cells.

The morphologies of crystals tended to be similar in microgravity and corresponding ground-control wells. Most crystals had sharp edges, although several wells contained crystals with plate or needle morphologies (Fig. 3 ▸ *a*). In wells 1 and 2, crystals grown in microgravity were larger and thicker than the crystals in parallel ground-control wells (Supplementary Fig. S2). Also, in well 29, ground-control samples grew only microcrystals from which no diffraction data could be collected, while crystals were larger in the ISS samples and diffraction data could be recorded. Conversely, crystals in well 35 grew larger on Earth than in microgravity, demonstrating that the size effect was not consistent between microgravity and Earth conditions. Microgravity-induced changes in crystal morphology have previously been reported (Takahashi *et al.*, 2013 ▸; Zörb *et al.*, 2002 ▸; Snell *et al.*, 1997 ▸; Savino & Monti, 1996 ▸); however, the morphologies that were observed in our experiments have all been observed previously for analogous crystals on Earth and did not represent new crystal forms.

Quantitative analyses of crystal number and visible crystal area from microscopy images demonstrated that fewer crystals of a size suitable for diffraction (>20 µm in each visible dimension) were grown per well in microgravity compared with ground controls (Fig. 3 ▸ *b*, Table 1 ▸). Two wells in each condition formed microcrystalline precipitate, microcrystals or stacks of needle crystals; these crystals were not included in crystal number and size analysis, which could have changed the data. Microgravity well 10 contained a large stack of needle crystals (∼250 crystals with longest edges of >20 µm) that could not be accurately counted, and microgravity well 35 contained ∼3000 microcrystals that were below the threshold of 20 µm in each visible dimension. In ground controls, well 29 contained ∼46 microcrystals and well 30 contained ∼230 microcrystals; these also were not included in the analysis.

Analyses of visible crystal area showed that the crystal size was larger in microgravity (Fig. 3 ▸ *b*, Table 1 ▸). Area was used for these analyses because only two dimensions were visible in each image. The largest microgravity-grown crystal was larger than the largest ground-control crystal. This agrees with previous reports of increased crystal size and decreased crystal number in microgravity (Abd Rahman *et al.*, 2015 ▸). Buoyancy-induced convection on Earth may increase the rate of nucleation in solutions containing crystals that are growing, termed secondary crystal nucleation, owing to a flow of partially nucleated proteins from growing crystal surfaces (Snell & Helliwell, 2005 ▸). Increased secondary nucleation would theoretically yield more and smaller crystals on Earth compared with microgravity, which is consistent with our findings.

We recorded images of crystals immediately upon receipt of experiments from the ISS, but no photographs of crystals could be taken in orbit during microgravity crystal growth owing to the incompatibility of the current ISS microscope hardware with the HDPGC growth cells, in particular owing to the variable opacity of the Chromex insert. To keep ground controls matched to microgravity experiments, ground controls were also not imaged during the six-month duration of the experiment. Thus, the optimal time for crystal nucleation and growth in microgravity is unclear. A different time frame may have produced more or larger crystals. Additionally, we found that crystals grew several months after return to Earth in two wells sent to the ISS that did not contain visible crystals upon initial return to Earth, and three-dimensional crystal growth also occurred in one well that had only irregularly shaped crystals upon initial return to Earth. This was confirmed by analysis of a second set of images taken of all crystallization wells five months after the experiment returned to Earth. Although crystal nucleation may have taken place on the ISS, we have categorized these as ‘ground’ crystals in Table 2 ▸ because most crystal growth occurred in a 1*g* environment. These ground crystals were not included in morphology, size or number analyses, but X-ray diffraction data were collected from several of these crystals.

### 3.2. Microgravity-grown crystals showed improved X-ray diffraction resolution on average, but the highest resolution and lowest mosaicity crystals grew on Earth   

Diffraction from microgravity and ground crystals was evaluated for resolution limit and mosaicity. High resolution is desirable to allow interpretation of the chemical details of a protein structure. Mosaicity is defined as the full-width at half-maximum of diffraction peaks. High average mosaicity values are a sign of a poorly ordered crystal and are generally undesirable because larger diffraction maxima can result in overlapping reflections. However, assessing mosaicity differences between crystals can be difficult owing to the requirement for the use of X-ray beams that have been conditioned to minimize spectral and geometric effects on the diffraction maxima.

MW1 was the only protein that crystallized in our experiments. MW1 Fab crystals were obtained in three space groups (Table 2 ▸). Crystals of MW1 Fab alone (space group *P*2_1_, unit-cell parameters *a* = 42, *b* = 72, *c* = 89 Å, β = 91°, one molecule per asymmetric unit) were obtained upon mixing MW1 Fab at 7 mg ml^−1^ with 0.1 *M* sodium citrate tribasic dihydrate pH 5.0, 18%(*w*/*v*) PEG 20 000. This condition yielded crystals that diffracted to 1.6–2.3 Å resolution, the highest resolution of any MW1 Fab crystals. These crystals formed after return from the ISS. Crystals that diffracted to 3.0 Å resolution were obtained in this space group and unit cell by mixing MW1 Fab at 7 mg ml^−1^ plus HD-16Q at 7 mg ml^−1^ with 0.2 *M* magnesium chloride hexahydrate, 0.1 *M* sodium citrate tribasic dihydrate pH 5.0, 10%(*w*/*v*) PEG 20 000 at 4°C, also in a well where crystal formation occurred after return from the ISS. Crystals did not form in microgravity or in ground controls in space group *P*2_1_, so the effect of microgravity on MW1 Fab crystals in this space group is unclear.

Crystals of MW1 Fab alone in a second space group (*P*2_1_2_1_2_1_, unit-cell parameters *a* = 442 *b* = 71, *c* = 208 Å, one molecule per asymmetric unit) were obtained upon mixing MW1 Fab at 7 mg ml^−1^ plus HD-39Q with one of four precipitant solutions at 4°C: (i) 0.1 *M* sodium acetate trihydrate pH 4.5, 30%(*w*/*v*) PEG 300, (ii) 1.8 *M* ammonium sulfate, 0.1 *M* bis-tris pH 6.5, 2%(*v*/*v*) PEG MME 550, (iii) 0.2 *M* magnesium chloride hexahydrate, 0.1 *M* sodium citrate tribasic dihydrate pH 5.0, 14%(*w*/*v*) PEG 20 000 or (iv) 0.2 *M* magnesium chloride hexahydrate, 0.1 *M* sodium citrate tribasic dihydrate pH 5.0, 18%(*w*/*v*) PEG 20 000. Over 70% of crystals that formed in microgravity or ground-control wells that diffracted to beyond 5.0 Å resolution belonged to this space group and unit cell. The highest resolution crystals in this space group were formed in ground controls; however, the average resolution improved by 0.2 Å and the average mosaicity of the diffraction data decreased by 0.03° (not statistically significant) in microgravity wells compared with ground-control wells. If the analysis is limited to only crystals that were looped immediately upon return from the ISS, the average resolution improved by 0.4 Å and the mosaicity decreased by 0.07° in microgravity wells compared with ground-control wells. This is consistent with previous findings of resolution improvements of 0.2–0.4 Å for crystals grown in microgravity compared with ground-control crystals (Strong *et al.*, 1992 ▸).

Crystals of MW1 in a third space group (*P*622, unit-cell parameters *a* = 189, *b* = 189, *c* = 64 Å, γ = 120°, one molecule per asymmetric unit) were obtained upon mixing MW1 Fab at 7 mg ml^−1^ with 0.1 *M* sodium citrate tribasic dihydrate pH 5.5, 16%(*w*/*v*) PEG 8000, or 0.1 *M* sodium citrate tribasic dihydrate pH 5.0, 18%(*w*/*v*) PEG 20 000 at 4°C. Crystals formed both in microgravity and in ground controls in this space group; however, the resolution was poor for all diffracting crystals, ranging from 3.2 Å to >8 Å. The highest resolution crystals were formed in microgravity, with an improvement of 0.8 Å in resolution and a decrease of 0.07° in mosaicity for the highest resolution microgravity crystal in this space group compared with the highest resolution ground-control crystal in the same space group. Ground-control crystals were 7 d fresher than microgravity crystals when they were cryo­preserved. Although unlikely over the total course of six months, it is possible that the results observed may have been affected by the seven-day difference in the duration of growth.

Previous studies have addressed whether particular space groups are more amenable to crystallization in a convection-free environment, and found that no space group appeared to be more amenable to improvement in microgravity (Judge *et al.*, 2005 ▸). Our results are consistent with this conclusion.

## 4. Discussion   

Microgravity affects crystal growth by decreasing buoyancy-driven forces on the crystal, thereby creating a more stable depletion zone around a growing crystal (Snell *et al.*, 2001 ▸). Microgravity also decreases crystal sedimentation, which leads to fewer fused aggregates and increased uniformity of crystals (Judge *et al.*, 2005 ▸). Through these mechanisms, microgravity-grown crystals have been reported to have increased resolution, decreased mosaicity and increased crystal volume compared with Earth-grown controls (Ng *et al.*, 1997 ▸; Barnes *et al.*, 2002 ▸); however, some negative studies have been published, and the benefit of microgravity crystallization has been fiercely debated (Stoddard *et al.*, 1992 ▸) since the first microgravity crystallization experiments more than 30 years ago (Littke & John, 1984 ▸).

Here, microgravity was used in an attempt to define the crystal structure of the polyQ repeat of huntingtin alone or bound to an anti-polyQ Fab (Hendricks *et al.*, 2009 ▸). While huntingtin was not crystallized in our experiments, crystals of the anti-polyQ Fab MW1 were readily obtained. Analysis of microgravity and Earth-grown MW1 Fab crystals showed that microgravity-grown crystals of MW1 Fab had an increase in size and an improvement in resolution and mosaicity on average when compared with Earth-grown crystals in one space group, in agreement with data published for other proteins (McPherson & DeLucas, 2015 ▸; DeLucas *et al.*, 1986 ▸); however, the highest overall resolution X-ray data in our experiments were obtained from a crystal grown on Earth after return from the ISS.

The observed increase in MW1 Fab crystal size in our microgravity experiments may have been driven by reduced buoyancy-induced convection in microgravity. However, these improvements in size may have also been owing in part to Marangoni convection and transient accelerations, which promote increases in crystal volume, despite possible deleterious effects on crystal packing (Kawaji *et al.*, 2003 ▸; Boggon *et al.*, 1998 ▸; Savino & Monti, 1996 ▸). Marangoni convection arises in vapor-diffusion experiments and occurs at the phase boundary between the concentrated solution of protein and the air. Concentration gradients that form during crystallization or precipitation result in differences in surface tension, which lead to different rates of transfer of vapor at the surface of the protein drop. While Marangoni convection is not the predominant method of mass transfer in crystallization experiments on Earth, it becomes an important factor when buoyancy-induced convection is substantially reduced in microgravity (Kawaji *et al.*, 2003 ▸; Chayen *et al.*, 1997 ▸). An analysis of microgravity experiments found greater improvements in crystal quality (X-ray diffraction resolution, signal-to-noise ratio and/or mosaicity) in liquid–liquid diffusion experiments compared with vapor-diffusion experiments (Judge *et al.*, 2005 ▸), which was hypothesized to be owing to decreased Marangoni convection in liquid–liquid diffusion compared with vapor-diffusion experiments. Our microgravity experiments were conducted using the HDPCG vapor-diffusion hardware; it is uncertain how much the use of a liquid–liquid diffusion apparatus would have impacted our results.

Transient accelerations on the ISS, such as residual accelerations from crew movement and exercising, vibrations imposed by equipment operating near crystallization experiments, and vehicle accelerations from reboost or collision-avoidance maneuvers (CAMs), could have led to deviation from a true microgravity environment. In a perfect microgravity environment, crystal nucleation occurs but growth is slowed because nutrients are depleted in the area of the crystal–solution interface. Brief accelerations may have stirred the solutions to replenish nutrients around crystals to help them grow larger. Previous research on the Space Shuttle with continuous visual feedback on crystal growth correlated increased crystal growth with increases in transient accelerations (Boggon *et al.*, 1998 ▸); no similar studies have been published for microgravity crystallization experiments on the ISS, so the impact of these transient accelerations is unclear.

While our experiments show that ISS is a potential platform for crystal growth, crystallization of proteins in space remains a challenge. Given the expense and time involved in crystallization trials in microgravity, future experiments should consider the potentially deleterious effects of Marangoni convection on vapor-diffusion crystallization experiments. Additionally, a comparison of microgravity-grown crystals with the best crystals obtainable through ground-based methods is necessary to realistically determine the relative value of microgravity protein crystallization.

## Supplementary Material

Supporting Information.. DOI: 10.1107/S2053230X16014011/rl5122sup1.pdf

## Figures and Tables

**Table 1:** Comparison of crystal number and size

Environment of crystals	No. of wells with crystals	Average No. of crystals per well† (range)	Mean crystal area‡ (µm^2^)	Largest crystal (µm^2^)
Microgravity	10	7 (5–13)	1840	42700
Ground control	9	14 (1–49)	1500	27200

† Average number of crystals >400 µm^2^ per well containing crystals.

‡ Geometric mean area.

**Table 2:** X-ray data-processing statistics for MW1 Fab crystals that diffracted to <5.0 Å resolution

		Overall resolution limit (Å)			Unit-cell parameters						
Environment of crystals	Well	CC_1/2_ > 0.3	〈*I*/σ(*I*)〉† > 1.50	Average mosaicity (°)	*a* (Å)	*b* (Å)	*c* (Å)	α (°)	β (°)	γ (°)	Space group
Microgravity	29	2.47	2.68	0.08	42.10	71.20	207.72	90	90	90	*P*2_1_2_1_2_1_
Microgravity	34	2.55	2.67	0.13	42.35	71.31	207.31	90	90	90	*P*2_1_2_1_2_1_
Microgravity	34	2.57	2.71	0.07	42.36	71.38	207.31	90	90	90	*P*2_1_2_1_2_1_
Microgravity	34	2.58	2.68	0.13	42.59	71.60	208.37	90	90	90	*P*2_1_2_1_2_1_
Microgravity	29	2.63	2.83	0.07	41.98	71.63	207.64	90	90	90	*P*2_1_2_1_2_1_
Microgravity	34	2.63	2.81	0.18	42.60	71.55	208.62	90	90	90	*P*2_1_2_1_2_1_
Microgravity	29	2.67	2.68	0.11	42.05	71.39	207.72	90	90	90	*P*2_1_2_1_2_1_
Microgravity	1	3.20	3.35	0.12	189.07	189.07	64.37	90	90	120	*P*622
Ground‡	2	1.59	1.71	0.06	42.28	71.62	89.19	90	91.51	90	*P*2_1_
Ground‡	2	1.65	1.80	0.26	42.21	72.19	89.92	90	91.95	90	*P*2_1_
Ground‡	2	1.72	1.80	0.19	42.19	71.53	89.08	90	90.96	90	*P*2_1_
Ground‡	2	1.87	2.03	0.09	42.23	71.69	89.05	90	91.39	90	*P*2_1_
Ground‡	2	2.19	2.32	0.06	42.19	71.61	88.88	90	91.34	90	*P*2_1_
Ground‡	2	2.29	2.61	0.08	42.26	71.70	89.04	90	91.59	90	*P*2_1_
Ground‡	17	3.00	3.00	0.15	42.48	72.37	89.78	90	91.43	90	*P*2_1_
Ground control	27	1.98	2.25	0.10	41.84	70.28	206.78	90	90	90	*P*2_1_2_1_2_1_
Ground control	27	2.13	2.45	0.06	41.94	70.43	207.36	90	90	90	*P*2_1_2_1_2_1_
Ground control	27	2.40	2.69	0.05	42.03	70.50	207.82	90	90	90	*P*2_1_2_1_2_1_
Ground control	35	2.72	2.88	0.07	42.35	71.30	207.37	90	90	90	*P*2_1_2_1_2_1_
Ground control	35	2.76	2.90	0.15	42.31	71.20	207.30	90	90	90	*P*2_1_2_1_2_1_
Ground control	35	2.76	2.95	0.34	42.37	71.10	208.32	90	90	90	*P*2_1_2_1_2_1_
Ground control	35	2.84	2.96	0.17	42.63	71.78	208.79	90	90	90	*P*2_1_2_1_2_1_
Ground control	27	2.89	2.98	0.16	42.22	71.01	208.95	90	90	90	*P*2_1_2_1_2_1_
Ground control	35	2.91	2.96	0.28	42.38	71.29	207.61	90	90	90	*P*2_1_2_1_2_1_
Ground control	27	3.96	3.70	0.06	41.90	70.43	207.75	90	90	90	*P*2_1_2_1_2_1_
Ground control	2	4.05	4.54	0.19	190.64	190.64	64.91	90	90	120	*P*6_2_22
Ground control	1	4.33	4.07	0.29	323.00	63.74	186.24	90	90	90	*P*2_1_2_1_2_1_

† 〈*I*/σ(*I*)〉 is the empirical signal-to-noise ratio (Karplus & Diederichs, 2012 ▸).

‡ Visible crystals grew in microgravity wells after return from the ISS.
