# Supplementary material for: Comparative analysis of anti-polyglutamine Fab crystals grown on Earth and in microgravity
Source: Acta Crystallogr F Struct Biol Commun. 2016 Sep 22;72(Pt 10):762–71. doi: 10.1107/S2053230X16014011 (PMC5053161; doi:10.1107/S2053230X16014011)
Supplement: Supplementary file 1 [file f-72-00762-sup1.pdf]

STRUCTURAL BIOLOGY COMMUNICATIONS

Volume 72 (2016)

Supporting information for article:

Comparative analysis of anti-polyglutamine Fab crystals grown on Earth and in microgravity

Gwen E. Owens, Danielle M. New, Alejandra I. Olvera, Julia Ashlyn Manzella, Brittney L. Macon, Joshua C. Dunn, David A. Cooper, Robyn L. Rouleau, Daniel S. Connor and Pamela J. Bjorkman

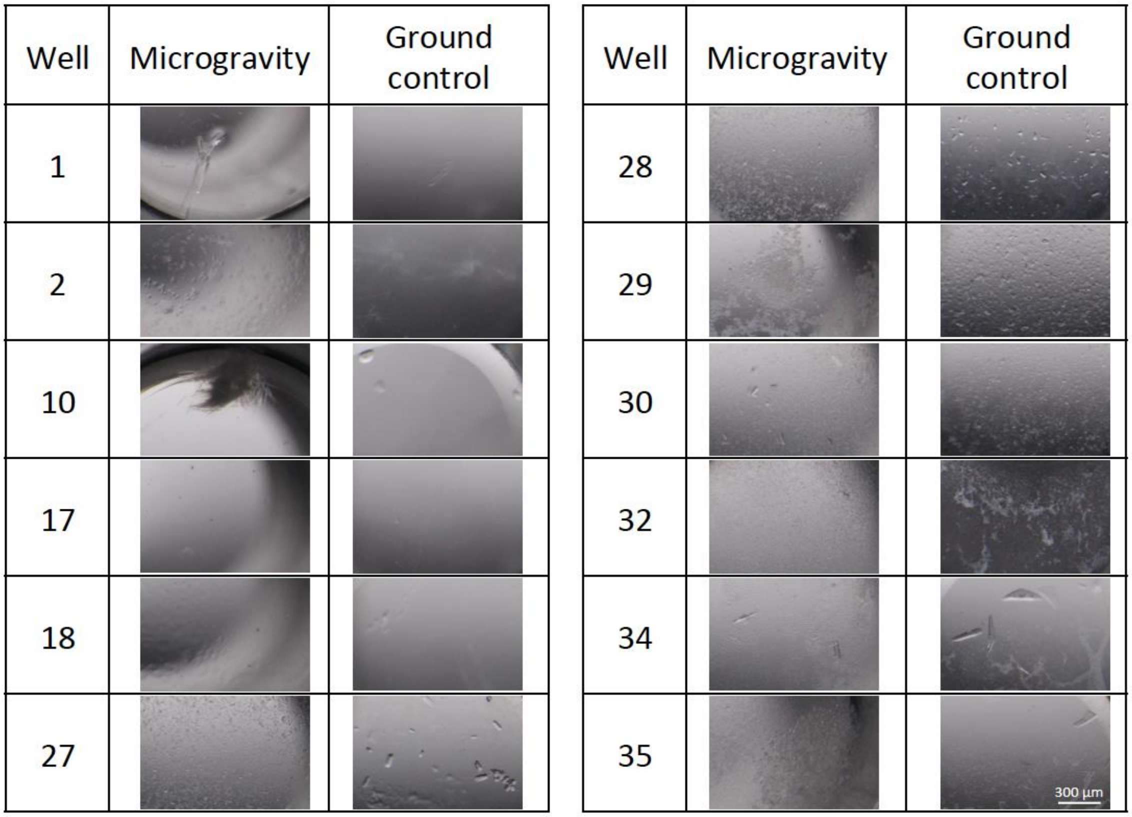

Figure S1 Representative images taken using bright field microscopy immediately upon completion of experiment. Crystals were observed in wells 1, 2, 8, 10 (microgravity only), 27-30, and 34-35. Crystals were not observed in wells 17, 18, and 32 immediately upon completion of experiment, as shown in figure.

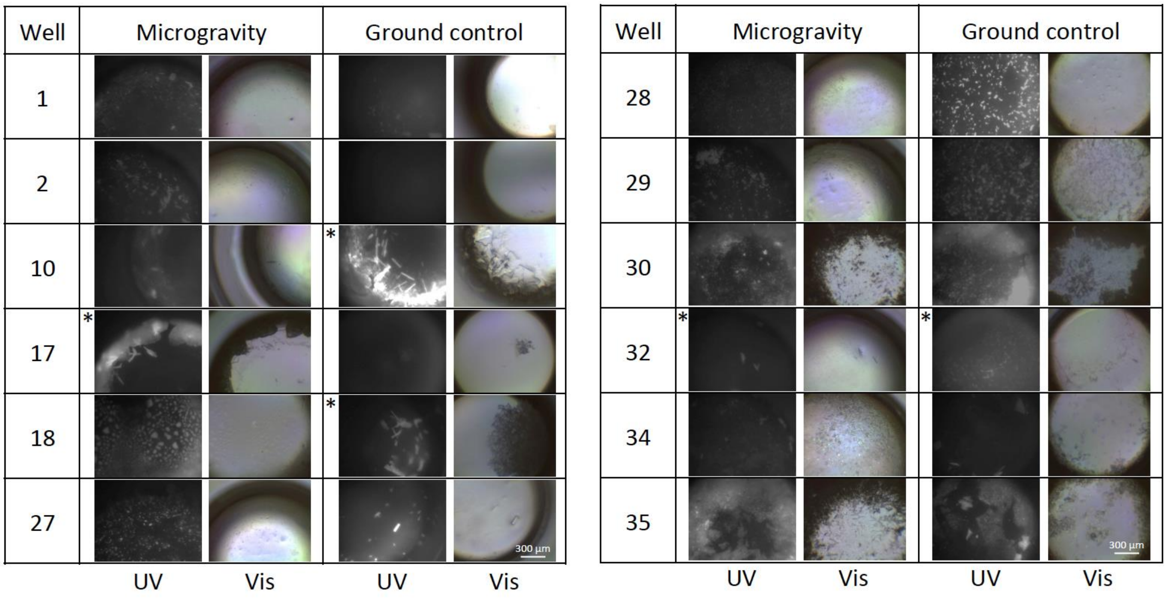

Figure S2 Representative crystal images taken using UV microscopy ten months after completion of experiment. *Crystals formed post-flight.

Table S1 Initial conditions for all microgravity crystallization experiments.

Table S1.
Initial conditions for all microgravity crystallization experiments.

Well	Protein Name(s)	Protein Conc (mg/mL)	Crystallization solution
1	MW1 Fab	7	0.1 M Sodium citrate tribasic dihydrate, pH 5.5, 16% w/v PEG 8,000
2	MW1 Fab	7	0.1 M Sodium citrate tribasic dihydrate, pH 5.0, 18% w/v PEG 20,000
3	MW1 Fab	7	0.2 M Ammonium citrate tribasic, pH 7.0, 0.1 M Imidazole, pH 7.0, 20% w/v PEG MME 2,000
4	3B5H10 Fab	7	0.2 M Magnesium chloride hexahydrate, 0.1 M Sodium citrate tribasic dihydrate, pH 5.0, 10% w/v PEG 20,000
5	3B5H10 Fab	7	1.8 M Ammonium sulfate, 0.1 M BIS-TRIS, pH 6.5, 2% v/v PEG MME 550
6	3B5H10 Fab + $K_2Q_{10}K_2$	7 + 7	1.8 M Ammonium sulfate, 0.1 M BIS-TRIS, pH 6.5, 2% v/v PEG MME 550
7	MW1 Fab + $K_2Q_{10}K_2$	7 + 7	0.1 M Imidazole, pH 7.0, 30% w/v PEG MME 550
8	MW1 Fab + $K_2Q_{10}K_2$	7 + 7	0.2 M Imidazole, pH 7.0, 28% w/v PEG MME 550
9	MW1 Fab + $K_2Q_{10}K_2$	7 + 7	0.2 M Magnesium chloride hexahydrate, 0.1 M Sodium citrate tribasic dihydrate, pH 5.0, 10% w/v PEG 20,000
10	MW1 Fab + $K_2Q_{10}K_2$	7 + 7	0.1 M Imidazole, pH 7.0, 12% w/v PEG 20,000
11	HD-16Q	5	0.1 M Tris-HCl, pH 8.5, 8% w/v PEG 8,000
12	HD-16Q	5	0.1 M Tris-HCl, pH 8.5, 8% w/v PEG 8,000
13	3B5H10 Fab + HD-16Q	7 + 7	0.2 M Magnesium chloride hexahydrate, 0.1 M Sodium citrate tribasic dihydrate, pH 5.0, 10% w/v PEG 20,000
14	3B5H10 Fab + HD-16Q	7 + 7	0.1 M Tris-HCl, pH 8.0, 30% v/v Jeffamine M-600, pH 7.0
15	3B5H10 Fab + HD-16Q	7 + 7	0.1 M Sodium acetate trihydrate, pH 4.0, 10% w/v PEG 4,000
16	3B5H10 Fab + HD-16Q	7 + 7	0.25 M Magnesium chloride hexahydrate, 0.1 M Sodium citrate tribasic dihydrate, pH 5.0, 15% w/v PEG 20,000
17	MW1 Fab + HD-16Q	7 + 7	0.2 M Magnesium chloride hexahydrate, 0.1 M Sodium citrate tribasic dihydrate, pH 5.0, 10% w/v PEG 20,000
18	MW1 Fab + HD-16Q	7 + 7	0.2 M Magnesium chloride hexahydrate, 0.1 M Sodium citrate tribasic dihydrate, pH 5.0, 18% w/v PEG 20,000
19	MW1 Fab + HD-16Q	7 + 7	0.1 M Imidazole, pH 7.0, 25% w/v PEG MME 550
20	MW1 Fab + HD-16Q	7 + 7	0.1 M Sodium citrate tribasic dihydrate, pH 5.0, 30% Jeffamine ED-2001 pH 7.0
21	MW1 Fab + HD-16Q	7 + 7	0.1 M BIS-TRIS, pH 6.5, 20% w/v PEG 1,500
22	MW1 Fab + HD-16Q	7 + 7	0.1 M Tris-HCl, pH 8.0, 28% w/v PEG 4,000
23	3B5H10 Fab + HD-39Q	7 + 2.3	0.1 M Sodium acetate trihydrate, pH 4.0, 15% w/v PEG 400
24	3B5H10 Fab + HD-39Q	7 + 2.3	0.1 M Sodium acetate trihydrate, pH 4.0, 15% w/v PEG 400
25	3B5H10 Fab + HD-39Q	7 + 2.3	0.1 M Sodium citrate tribasic dihydrate, pH 5.0, 18% w/v PEG 20,000
26	3B5H10 Fab + HD-39Q	7 + 2.3	0.1 M Sodium citrate tribasic dihydrate, pH 5.0, 18% w/v PEG 20,000
27	MW1 Fab + HD-39Q	7 + 2.3	0.1 M Sodium acetate trihydrate, pH 4.5, 30% w/v PEG 300
28	MW1 Fab + HD-39Q	7 + 2.3	0.1 M Sodium acetate trihydrate, pH 4.5, 30% w/v PEG 300
29	MW1 Fab + HD-39Q	7 + 2.3	1.8 M Ammonium sulfate, 0.1 M BIS-TRIS, pH 6.5, 2% v/v PEG MME 550
30	MW1 Fab + HD-39Q	7 + 2.3	0.2 M Magnesium formate dihydrate, 0.1 M Sodium acetate trihydrate, pH 4.0, 18% w/v PEG MME 5,000
31	MW1 Fab + HD-39Q	7 + 2.3	0.1 M Sodium citrate tribasic dihydrate, pH 5.5, 18% w/v PEG 3,350
32	MW1 Fab + HD-39Q	7 + 2.3	0.1 M Sodium citrate tribasic dihydrate, pH 5.5, 16% w/v PEG 8,000
33	MW1 Fab + HD-39Q	7 + 2.3	2% w/v 1,4-Dioxane, 0.1 M Tris-HCl, pH 8.0, 15% PEG 3,350
34	MW1 Fab + HD-39Q	7 + 2.3	0.2 M Magnesium chloride hexahydrate, 0.1 M Sodium citrate tribasic dihydrate, pH 5.0, 14% w/v PEG 20,000
35	MW1 Fab + HD-39Q	7 + 2.3	0.2 M Magnesium chloride hexahydrate, 0.1 M Sodium citrate tribasic dihydrate, pH 5.0, 18% w/v PEG 20,000
36	MW1 Fab + HD-39Q	7 + 2.3	0.4 M Sodium malonate, pH 6.0, 0.1 M MES monohydrate, pH 6.0, 0.5% w/v PEG 10,000
37	HD-16Q	10	0.2 M Ammonium acetate, 0.1 M Sodium citrate tribasic dihydrate, pH 5.5, 30% w/v PEG 4,000
38	HD-16Q	10	0.2 M Magnesium acetate tetrahydrate, 0.1 M Sodium cacodylate trihydrate, pH 6.5, 20% w/v PEG 8,000
39	HD-16Q	10	0.5 M Ammonium sulfate, 0.1 M Sodium citrate tribasic dihydrate, pH 5.6, 1.0 M Lithium sulfate monohydrate
40	HD-16Q	10	0.2 M Magnesium chloride hexahydrate, 0.1 M Tris-HCl, pH 8.5, 25% w/v PEG 3,350
41	HD-25Q	10	0.01 M Nickel (II) chloride hexahydrate, 0.1 M Tris-HCl, pH 8.5, 20% w/v PEG MME 2,000
42	HD-25Q	10	0.01 M Nickel (II) chloride hexahydrate, 0.1 M Tris-HCl, pH 8.5, 1.0 M Lithium sulfate monohydrate
43	HD-25Q	10	1.0 M Imidazole, pH 7.0
44	HD-25Q	10	0.1 M BIS-TRIS, pH 6.5, 20% w/v PEG MME 5,000
45	HD-39Q	10	0.01 M Iron (III) chloride hexahydrate, 0.1 M Sodium citrate tribasic dihydrate, pH 5.6, 10% Jeffamine M-600
46	HD-39Q	10	1.0 M Imidazole, pH 7.0
47	HD-46Q	10	0.2 M Calcium acetate hydrate, 0.1 M Sodium cacodylate trihydrate, pH 6.5, 18% w/v PEG 8,000
48	HD-46Q	10	0.5 M Sodium chloride, 0.01 M Magnesium chloride hexahydrate, 0.01 M Hexadecyltrimethylammonium bromide
49	HTT-GFP	20	0.2 M Zinc acetate dihydrate, 0.1 M Sodium cacodylate trihydrate, pH 6.5, 18% w/v PEG 8,000
50	HTT-GFP	20	0.2 M Zinc acetate dihydrate, 0.1 M Sodium cacodylate trihydrate, pH 6.5, 18% w/v PEG 8,000
51	HTT-GFP	20	0.2 M Zinc acetate dihydrate, 0.1 M Sodium cacodylate trihydrate, pH 6.5, 18% w/v PEG 8,000
52	HTT-GFP	20	0.2 M Lithium sulfate monohydrate, 0.1 M HEPES, pH 6.5, 25% w/v PEG 3,350
53	HTT-GFP	20	0.2 M Lithium sulfate monohydrate, 0.1 M HEPES, pH 6.5, 25% w/v PEG 3,350
54	HTT-GFP	20	0.2 M Lithium sulfate monohydrate, 0.1 M HEPES, pH 6.5, 25% w/v PEG 3,350
55	FL-HTT	0.4	0.1 M Sodium cacodylate trihydrate, pH 6.5, 25% w/v PEG 8000
56	FL-HTT	0.4	0.1 M Sodium cacodylate trihydrate, pH 6.5, 25% w/v PEG 8000
57	FL-HTT	0.4	2.4 M Ammonium sulfate, 0.1 M Sodium acetate trihydrate, pH 4.5
58	FL-HTT	0.4	2.4 M Ammonium sulfate, 0.1 M Sodium acetate trihydrate, pH 4.5
59	FL-HTT	0.4	2.4 M Ammonium sulfate, 0.1 M Sodium cacodylate trihydrate, pH 6.5
60	FL-HTT	0.4	2.4 M Ammonium sulfate, 0.1 M Sodium cacodylate trihydrate, pH 6.5

Table S2 Crystal morphology.

Table S2.
Crystal morphology

Environment of crystals	Well	Morphology
Microgravity	1	3D
Microgravity	2	Irregular
Microgravity	8	Needle
Microgravity	10	Needle
Microgravity	17	Grew post-flight
Microgravity	27	3D
Microgravity	28	3D
Microgravity	29	3D
Microgravity	30	3D
Microgravity	32	Grew post-flight
Microgravity	34	3D
Microgravity	35	3D
Ground control	1	Irregular
Ground control	2	Needle
Ground control	8	Irregular
Ground control	10	Grew post-flight
Ground control	18	Grew post-flight
Ground control	27	3D
Ground control	28	3D
Ground control	29	3D
Ground control	30	3D microcrystals
Ground control	32	Grew post-flight
Ground control	34	3D
Ground control	35	3D
